# Aligned 3D porous polyurethane scaffolds for biological anisotropic tissue regeneration

**DOI:** 10.1093/rb/rbz031

**Published:** 2019-09-30

**Authors:** Weiwei Lin, Wanling Lan, Yingke Wu, Daiguo Zhao, Yanchao Wang, Xueling He, Jiehua Li, Zhen Li, Feng Luo, Hong Tan, Qiang Fu

**Affiliations:** 1 College of Polymer Science and Engineering, State Key Laboratory of Polymer Materials Engineering, Sichuan University, Chengdu 610065, China; 2 Sichuan Institute for Food and Drug Control, Chengdu 611731, China; 3 Department of Neurosurgery West China Hospital, Sichuan University, Chengdu 610065, China; 4 Laboratory Animal Center of Sichuan University, Chengdu 610041, China

**Keywords:** polyurethane, aligned scaffolds, tissue engineering, anisotropic regeneration

## Abstract

A green fabrication process (organic solvent-free) of artificial scaffolds is required in tissue engineering field. In this work, a series of aligned three-dimensional (3D) scaffolds are made from biodegradable waterborne polyurethane (PU) emulsion via directional freeze–drying method to ensure no organic byproducts. After optimizing the concentration of polymer in the emulsion and investigating different freezing temperatures, an aligned PUs scaffold (PU14) generated from 14 wt% polymer content and processed at −196°C was selected based on the desired oriented porous structure (pore size of 32.5 ± 9.3 μm, porosity of 92%) and balanced mechanical properties both in the horizontal direction (strength of 41.3 kPa, modulus of 72.3 kPa) and in the vertical direction (strength of 45.5 kPa, modulus of 139.3 kPa). The response of L929 cells and the regeneration of muscle tissue demonstrated that such pure material-based aligned 3D scaffold can facilitate the development of orientated cells and anisotropic tissue regeneration both *in vitro* and *in vivo*. Thus, these pure material-based scaffolds with ordered architecture have great potentials in tissue engineering for biological anisotropic tissue regeneration, such as muscle, nerve, spinal cord and so on.

## Introduction

Many biological tissues *in vivo*, such as muscle, tendon and nerve, contain the extracellular matrix (ECM) with highly aligned architecture and anisotropic mechanical properties [1, 2]. Such ECM provides a physical guide for cells to form an anisotropic aggregation. Tissue engineering is developed to repair the injured tissues via mimicking the ECM, or rebuilding the ECM [3]. Based on this opinion, tremendous efforts have been made to endow substrates with ordered structure in the aligned tissue reconstitution. For example, Liu et al. prepared an orientated electrospun mesh which could support the growth of human dermal fibroblasts better compared with random mesh [4]. Most reports about the aligned control of cells focus on two dimensional substrates. However, cells actually proliferate and migrate in three-dimensional (3D) environment *in vivo* [5].

The porous architecture and matched mechanical properties of tissue regeneration scaffolds are two crucial factors to rebuild natural ECM [6]. It was proved that angiogenesis, biodegradation rate, growth factors inflow and other biological properties depend significantly on the pore structure of the scaffolds [7]. For example, Liu et al. well facilitated the new bone regeneration with a shape memory porous scaffold [8]. In addition, the mechanical anisotropy of the scaffolds caused by orientation will certainly limit random cell motility and polarity [9]. Contact guidance demonstrates that cells will choose a path with less barriers to migrate [10]. Thus, the topography of substrates will impact the behaviors of cells. A scaffold with an anisotropic structure may offer a chance to rebuild the damaged tissues. As described in literature, the channel-like pores can act as a guiding structure for ordered ECM reconstruction [11].

Freeze–drying method is thought to be an eco-friendly and economical way to prepare 3D porous scaffold, because it uses no organic solvent and produces no byproducts except water compared with other methods like electrospinning and solution casting/particulate leaching [12,13]. With the help of ‘self-cleaning’ nature of ice, water excludes the solutes to form ice crystal. By tuning emulsion concentration and freezing temperature, the size and distribution of ice can be controlled [14]. Then by means of lyophilization, interconnecting porous structure forms after ice being removed. Based on the freeze–drying method, directional freezing is put up forward to construct aligned architectures. The temperature gradient are applied in one certain direction of samples, and regular ice columns come into being [15]. With the directional freezing method, many kinds of water-soluble polymers were applied to prepare aligned porous scaffolds, such as poly(vinyl alcohol), poly-L-lactide/ poly(ethylene glycol) (PEG) blend and so on [16,17]. The ordered structures obtained from this method play important roles in different fields. For example, Zeng et al. applied directional freezing to construct 3D networks in polymer composites for the improvement of thermal conductivity and Kota et al. reported ice-templated 3D nitrogen-doped graphene for the enhancement of supercapacitor performance [18,19]. It is worth noting that directional freezing is not fully developed in tissue engineering. With the help of directional freezing, Francis et al. reported that the aligned chitosan-alginate scaffold can induce the extended neurites of dorsal root ganglion (DRG) to grow along the channels [20], and Asuncion et al. observed the bone marrow stem cells can arrange along the orientated silk fibroin/gelatin scaffold [21]. Moreover, most of the attempts were performed *in vitro*. The performance of the 3D aligned structure constructed by the directional freezing method is rarely checked *in vivo*.

Polyurethanes (PUs) are common polymeric materials used in medical implant applications, in part due the ability to design their biocompatibility and achieve tunable mechanical properties [22–25]. In our previous work, a series of waterborne biodegradable PUs emulsion have been synthesized. Song et al. used isophorone diisocyanate, poly(e-caprolactone) (PCL), PEG, 1,4-butandiol and l-lysine to obtain a non-toxic PU for cultivating bladder smooth muscle cells, endothelial cells and fibroblasts [22]. Hao et al. designed a lysine-based PU emulsion using PCL and PEG as soft segments, l-lysine ethyl ester diisocyanate (LDI), 1,3-propanediol (PDO) and l-lysine derived from human metabolism products as hard segments [27]. The scaffolds made from emulsion were implanted into rats’ traumatic brain injury model and performed well [28]. Based on the satisfactory performance of PU emulsion showed above, great interest arouses to develop aligned 3D porous scaffolds for ordered tissue repair by using such PU emulsion.

In this work, biodegradable waterborne PU scaffolds with aligned porous structures were prepared through directional freeze–drying method. By tuning emulsion concentration and freezing temperature, a series of pure material-based scaffolds with different ordered architectures were obtained. The morphology of scaffolds was observed by scanning electron microscopy (SEM). The tensile mechanical tests were applied to study their anisotropic mechanical properties. To identify the biological ability of the aligned porous scaffolds, L929 cells were planted in these scaffolds. And then the optimal PU scaffolds were implanted into the leg muscle of rat. The immunofluorescent staining and immunohistochemical characteristics were employed to confirm the effects of the aligned 3D porous scaffold on the cell behaviors and the accelerated tissue regeneration of muscle.

## Materials and methods

### Materials

LDI (Nantong Dahong Chemical industry limited company) and PDO (Chengdu Kelong Reagent Co.) were redistilled under vacuum before use. PEG (molecular weight 1450, Dow Chemical) and PCL (molecular weight 2000, Dow Chemical) were dehydrated at 95–100°C under vacuum for 2 h. The chain extender l-lysine was used as received. Organic bismuth catalyst was BiCAT (The Shepherd Chemical Company). L929 cells were obtained from West China Medical Center of Sichuan University (Sichuan, China). Dulbecco’s modified Eagle’s medium (DMEM) was purchased from Gibco Life (Grand Island, NY). Fetal bovine serum (FBS) was purchased from Hyclone (Logan, UT). Rhodamine phalloidin was purchased from Invitrogen. Anti-FAK (phospho Y397), anti-desmin and anti-muscle actin were purchased from Abcam.

### Synthesis of PU

PU emulsion was synthesized according to our previous report and the synthetic procedure is schematized in Supplementary Figure S1 [27]. Briefly, LDI and 0.1% organic bismuth (catalyst) were added to agitated PCL and PEG at 80°C under protection of dry nitrogen, following by the addition of chain extender PDO at 65°C for 2 h. In sequent process, the pre-polymer was poured into l-lysine solution under agitation at high speed for the second chain extending. The reaction of emulsification was carried out for 3 h with dropwise addition of dilute sodium hydroxide solution to adjust pH to 8. In the PU emulsion, the molar ratio of LDI, PEG, PCL, PDO, L-lysine is 3.6:0.25:0.75:1:1 (NCO/(OH+NH2)=1.2). PCL and PEG are soft segments. PDO and l-lysine are chain extenders. LDI is the hard segment. NaOH is used to expose amino groups of l-lysine, making it easier to react with NCO groups. At the same time, NaOH changes –COOH into –COO-, contributing to better hydrophilicity.

### Preparation of porous scaffolds

**Figure 1:**
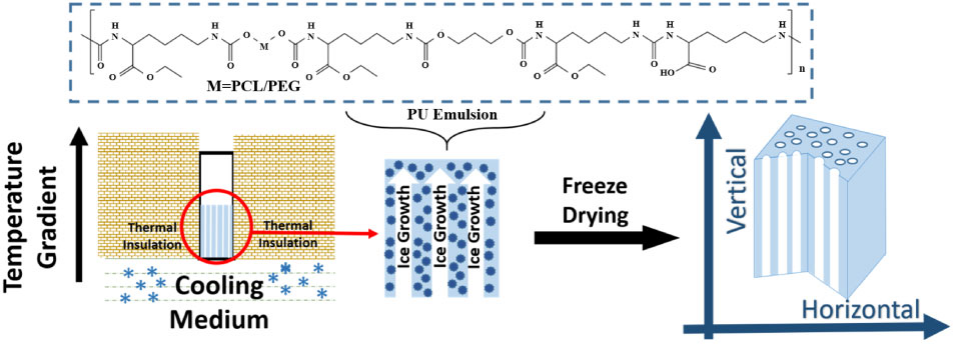
The fabrication process of aligned 3D porous PU scaffolds using biodegradable waterborne PU emulsion by directional freeze–drying method

Aligned 3D porous scaffolds were prepared by using directional freeze–drying method as shown in Fig. 1. Briefly, different concentrations (6, 10 and 14 wt%, adjusting by distilled water) of PU emulsion at the volume of 400 μl were poured in to the cubic molds of polystyrene, followed by treatment in a single temperature gradient direction until ice coming into being (about 30 min). The liquid nitrogen (LN) was taken as a frozen medium for −196°C. Mixture with specific proportion of alcohol and LN can achieve frozen medium for −40 and −80°C, respectively. All freezing medium were placed under the molds, and all of them did not directly contact with the PU emulsion. After above operation, the emulsion of different concentration and freezing temperature in the mold were dealt with lyophilization and aligned porous structures were obtained.

Random porous scaffolds: concentration of 14 wt% of emulsion at the volume of 400 μl was poured in to the cubic molds of polystyrene, followed by being stored in 4°C for 4 h and then stored in −20°C environment for 24 h to allow ice coming into being. After above operation, the emulsion in the mold was dealt with lyophilization and random porous structure was obtained.

### Preparation of PU films

Emulsion at the concentration of 20 wt% was poured into a round mold of tetrafluoroethylene and then was stored in a chamber at 40°C for 24 h to evaporate water. After the PU film were prepared, the films were cut into a dimension of 5 mm × 5 mm.

### Scanning electron microscopy

The morphology of the porous scaffolds was investigated by SEM (FEI, Quanta 200, Philips, Netherlands) at 20 kV after gold coating. Micrographs were digitized and analysed with Image J to determine the average pore diameter and porosity of the porous scaffolds.

### Tensile testing

The tensile measurements were performed on a commercial tensile tester (HZ-1004 Dongguan lixian instrument Scientific Co. Ltd) at a stretching rate of 10 mm min^−1^ at room temperature. Both vertical and horizontal directions of the scaffolds were tested. The porous scaffolds were all in a dimension of 0.2 cm × 1 cm × 4 cm. Initial modulus was calculated from the stress–strain curve as the slope of the initial linear portion of the curve. All of the values were expressed as the mean ± standard deviation (*n* ≥ 3).

### Cell culture

L929 cells were obtained from West China Medical Center of Sichuan University (Sichuan, China), and cultured in complete DMEM (Gibco Life, Grand Island, NY, USA) supplemented with 10% FBS (Hyclone, Logan, UT), 100 units/ml penicillin and 100 units/ml streptomycin (Gibco) at 37°C. The entire cell culture system was kept in air containing 5% carbon dioxide and at approximately 90% relative humidity in culture bottles. The medium was renewed every 2 days. Cells in increased logarithmic phase were rinsed with sterilized PBS and then incubated in 0.25% trypsin for 3 min. Trypsinization was stopped by adding DMEM medium with 10% FBS. Cells were centrifuged and resuspended in DMEM with 10% FBS and then diluted into certain concentration.

Before cell seeding, porous scaffolds were sterilized with gamma radiation using doses of 25 kGy. Then the liquid was removed from the wells. After two passages, cells reached confluence and were removed by trypsin treatment, counted and seeded on WBPU films and porous scaffold at a density of 50 000 cells/well. The cellular constructs were maintained in an incubator at 37°C.

### Immunofluorescent staining

Cells on the films and 3D porous scaffolds were first fixed with paraformaldehyde (4% (w/v)) in 37°C for 20 min and the cell membrane was permeabilized by Triton X-100 (0.8% (v/v)) for 10 min. After cells were blocked by BSA (5% in PBS (w/v)) at room temperature for 1 h, the primary antibodies were added and incubated at 4°C overnight. Then secondary antibodies (green) were added and incubated at room temperature for 1 h. After that, the F-actin and nuclei were stained with Rhodamine Phalloidin (red) and DAPI (blue), respectively. Finally, fluorescent images were taken with a laser scanning confocal microscopy (Nikon N-SIM).

### Animal model

Under the guidance of the Principles of Laboratory Animal Care of the National Institute of Health, China, the animal model was built up after being approved by the Ethics Committee of Sichuan University. The animal approval number is SCXK (chuan) 2013-026. SPF adult Sprague Dawley (SD) female rats (Institute of Laboratory Animal of Sichuan Academy of Medical Sciences, Chengdu, China) were employed in this study. All rats at the age of 8 weeks fed with standard laboratory chow and distilled water administered were kept in 20–22°C temperature and 50–60% relative humidity. 12 h artificial light-dark cycles were applied to them. Before surgery, operation instruments were disinfected and rats were injected with 3% pentobarbital sodium of 0.3% weight. The hair covering their both legs were removed. All rats were implant random porous scaffolds in the incision of 1 cm on left leg and aligned scaffolds in the incision of the same size on the right legs after being carefully disinfected (*n* = 4). And the incision of both left and right legs were closed. Muscle recovery of left legs with random scaffolds and right legs with aligned scaffolds would be compared. They were sacrificed at the timing of 1 and 2 weeks.

After rat were sacrificed, leg muscle tissues in the range of 5 cm around implanted scaffolds were collected and frozen in an O.C.T. (SAKURA, Japan) embedding medium at −80°C. About 5 μm sections were obtained via microtome (Leica, Germany) and were stained with hematoxylin and eosin. For immunohistochemical characteristics, sections of implanted scaffolds and muscle tissues were stained with desmin and muscle-actin as manufacturer's instructions. Light microscope (Leica, Germany) were applied to observe the sections.

### Statistical analysis

The average size, distribution and alignment of the aligned 3D porous scaffolds were collected from SEM images (*n* ≥ 6) and were semi-quantified by Image J. Porosity was calculated from the equation: Porosity =$100\%*\frac{\left[V\left(\mathrm{Scaffold}\right)-V\left(\mathrm{film}\right)\right]}{V\left(\mathrm{Scaffold}\right)}$,  where V is the Volume (*n* ≥ 3). All data (except distribution data) are represented as mean values with ± standard deviations (SD).

## Results and discussion

### Preparation and morphologies of aligned porous PU scaffolds

As shown in Fig. 1, thanks to the emulsion state of waterborne PU, the directional freezing method is not only feasible but also clean to prepare such pure PU material-based 3D porous scaffolds. Herein, biocompatible PU emulsion was synthesized as our previous report [27]. By tuning the concentration of emulsion and freezing temperature, a series of PU scaffolds with different architectures and mechanical properties were obtained. According to the concentration of PU from 6 to 14 wt%, the aligned scaffolds are named as PU6, PU10 and PU14, respectively. Random porous PU scaffolds with concentration of 6, 10 and 14 wt% are also fabricated by normal freeze–drying without directional freezing procedure. Further, in order to highlight the guiding function of aligned scaffolds for the regeneration of ordered tissue, random porous PU scaffold of 14 wt% is used as a control sample in cell culture and tissue response assessment.

**Figure 2:**
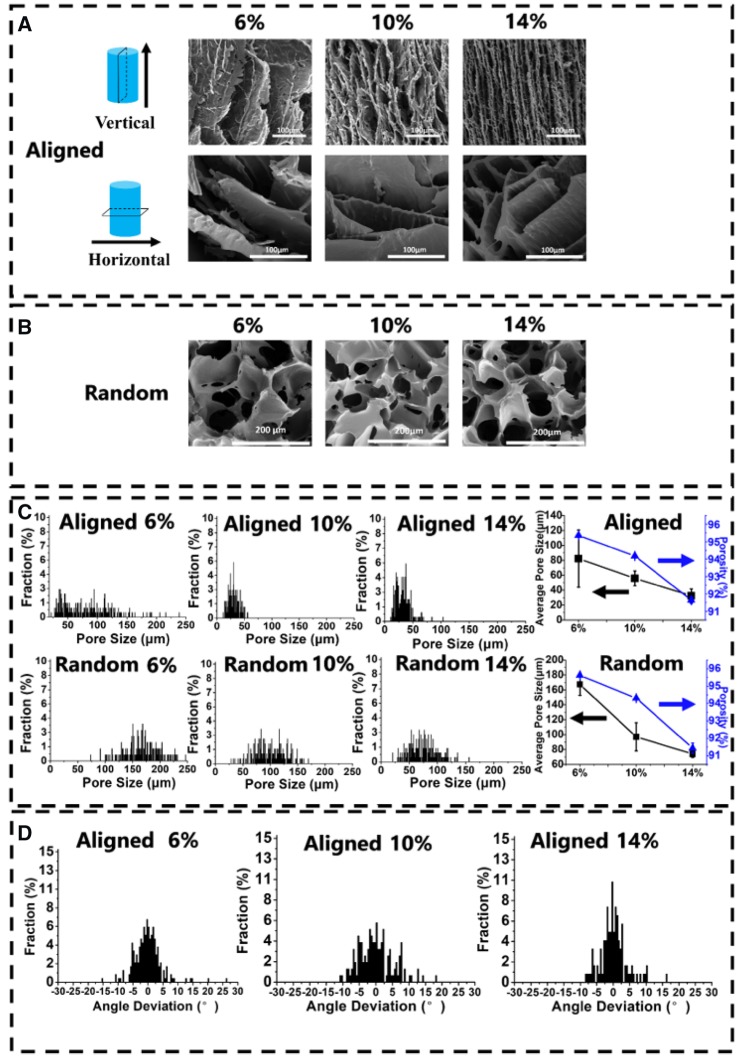
(**A**) SEM photographs of aligned porous PU scaffolds with different concentrations (6, 10 and 14 wt%) processed at −196°C. (**B**) SEM photographs of random porous PU scaffolds with different concentrations. (**C**) The distribution of pore size, average pore size and porosity of aligned and random scaffolds with different concentrations. (**D**) Histogram of the alignment in aligned scaffolds with different concentrations

As shown in Fig. 2A, when the concentration of PU emulsion increased, the microstructures of the scaffolds varied from ‘layer’ like structure to ‘fish-bone’ like structure with the pore size decreasing from 82.3(±38.3) to 32.5(±9.3) μm. The change of structure is possibly because of the perturbation happened on the interfaces between solutes and water [29]. The extent of perturbation depends on behaviors of ice exclusion. When the emulsion with relatively high concentration rapidly freezes at −196°C, the dendritic and coarse morphology of the scaffolds forms [30], so that smooth ‘layer’ structure changes into rugged ‘fish-bone’ structure. And the tendency of pore size for aligned scaffolds meets the same trend of the random scaffolds (Fig. 2B). When emulsion with low concentration forms a scaffold, water with nature of so-called ‘self-cleaning’ excludes solutes to translate into thick icicles leaving large pores after freeze–dried process. Furthermore, there is still no golden standard for the most suitable pore size for cell ingrowth [31, 32]. In other words, ideal pore size may vary with different cells and materials. The smallest pore size is considered to range from 10 to 50 μm. Fortunately, most of the pores in our aligned scaffolds with 10 and 14 wt% concentration are in this range. Due to the different freezing procedure, the random scaffolds have the larger pore size over 50 μm. Meanwhile, increasing concentrations make a slight decline in porosity (Fig. 2C), but the porosity of all random and aligned scaffolds are more than 90%. In addition, the aligned scaffold structure became more regular in concentration of 14 wt% compared with other two aligned scaffolds (6 and 10 wt%) (Fig. 2D).

For consideration of the directional freezing temperature, three freezing temperature −40, −80 and −196°C (LN) were set for samples at a fixing concentration of 14 wt%. As shown in Supplementary Figure S2, with temperature decreasing, the smooth layer of PU14-(−40°C) scaffold turned into ‘fish-bone’ structure of PU14-LN scaffold, and the size of pores were also decreased. The lower freezing temperature, the faster ice crystals grow and the smaller ice columns form [33]. After being dried, the pores certainly will be smaller in lower freezing temperature. In contrast, the water in the emulsion freezed at relatively high freezing temperature (−40 and −80°C) would undergo a longer time to form more perfect ice crystals. Hence, the fluctuation at the interfaces between solutes and water decreased [14], and then a relatively smooth ‘layer’ formed. However, it has been reported that the rough substrate would be in favor of the cell adhesion and cell growth because of the fibrous reticular morphology of natural ECM [34]. Considering the structural regularity and pore size, the optimal freezing temperature of aligned PU scaffolds are selected at −196°C. Therefore, all the following aligned PU scaffolds are prepared at freezing temperature of −196°C.

### Mechanical properties tests

**Figure 3:**
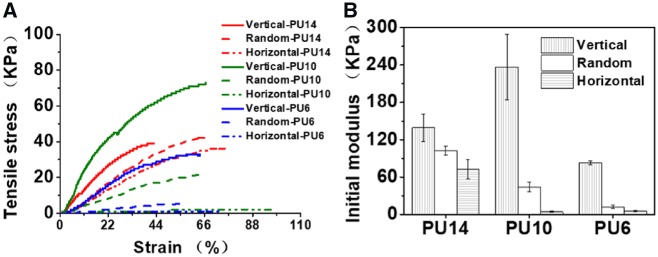
(**A**) Tensile curves of the porous PU scaffolds with different concentrations processed at −196°C. (**B**) Initial modulus of the porous PU scaffolds with different concentrations processed at −196°C

The anisotropic mechanical properties of the porous PU scaffolds are shown in Fig. 3. Ordered structure endows the scaffolds different mechanical properties in vertical and horizontal direction. As shown in Fig. 3A, the tensile strengths of the aligned PU scaffolds at vertical direction are always stronger than those in the horizontal direction. The tensile strengths of aligned scaffolds in vertical direction vary from 36.5 to 71.5 kPa with the polymer concentration, while these strengths in horizontal direction vary from 2.3 to 41.3 kPa. And no matter what concentrations, aligned scaffolds exhibit higher initial modulus in the vertical direction than that in the horizontal one (Fig. 3B), similarly as the previous reports [21]. In vertical directions, solutes built continuous ‘layer’ or ‘fish-bone’ structure compared with the discontinuity in the horizontal directions. In addition, the initial modulus of random scaffolds is always intermediate, which is below the vertical one but above the horizontal one with the same concentration. Due to the ‘fish-bone’ structure of the aligned PU14, the initial modulus of aligned PU14 (72.3(±15.2) kPa) in horizontal direction is significantly greater than those of PU10 (4.0(±0.8)) and PU6 (5.3(±0.8) kPa). With consideration of the modulus in both directions, the aligned PU14 exhibited relatively balanced mechanical properties (strength of 41.3 kPa, modulus of 72.3 kPa in the horizontal direction and strength of 45.5 kPa, modulus of 139.3 kPa in the vertical direction). Such mechanical properties and smaller gap between the vertical and horizontal modulus of PU14 could enhance the resistance of deformation as well as preserve its anisotropy *in vivo*.

As a result of suitable morphology and tough mechanical properties, the aligned 3D porous scaffold of PU14 is superior to others in cell culture *in vitro* and tissue response *in vivo* as demonstrated below.

### Cellular response to aligned scaffolds

**Figure 4:**
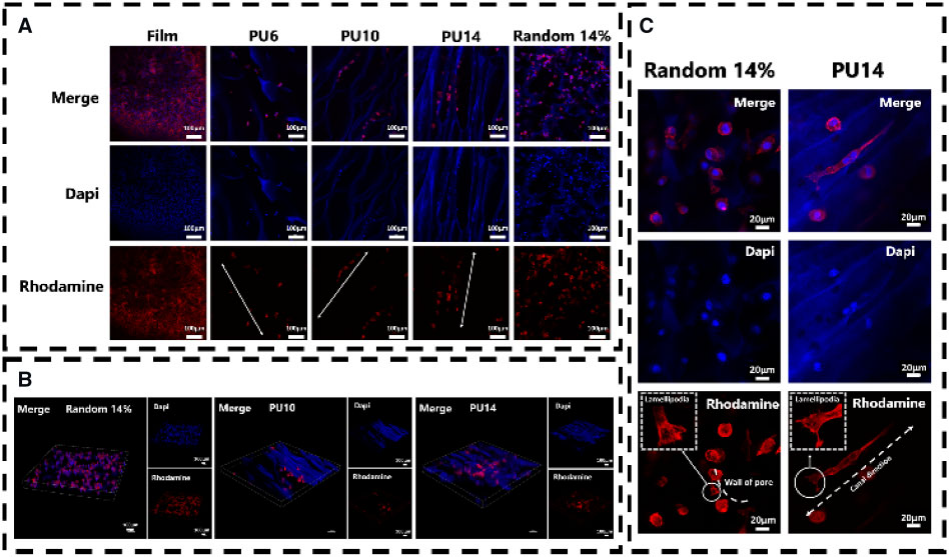
(**A**) Confocal microscope images of L929 cells cultured for 8 days on PU film and scaffolds. The arrows in the pictures are the oriented canal direction. (**B**) Combined image of optically sectioned confocal microscope images of scaffolds (random 14%, PU10 and PU14). (**C**) Confocal microscope images of L929 cells cultured for 8 days on the random porous scaffold and the aligned porous scaffold of PU14. Nuclei of cells were stained with dapi, blue; F-actin of cells were stained with rhodamine, red

L929 cell, a kind of fibroblast, was chosen as cell model *in vitro*, because it can perform an orientated morphology under some certain conditions, such as on the ordered substrate [35, 36]. As shown in Fig. 4A, murine L929 cells could fully extend themselves on the PU substrates either film or scaffolds after 8 days cultured. This indicates that the biocompatible PU could facilitate L929 cells to survive and gain appropriate morphology. Particularly, L929 can be seen in the pores (or called as ‘canals’) of PU6, PU10 and PU14 as well as random porous PU scaffolds (random 14%). In addition, because PU can be stained with dapi (blue), the structure of scaffolds can be also identified in confocal photos. Unlike on PU film and random 14%, L929 cells prefer to queue along with the orientated canals in the aligned PU6, PU10 and PU14. Compared with cells cultured for 3 days in Figure S3, L929 cells cultured for 8 days increased in amount, and they overspread the PU film and random 14% sample with isotropic clutters. The number of cells in random 14% is even more than that in the aligned PU14. However, cells in the PU14 is aligned and proliferate better than the PU6 and PU10. Moreover, the cells in the scaffolds are further observed by combined image of optically sectioned confocal microscope images (Fig. 4B). Since there are a few cells on the PU6, the PU6 was excluded from combined image. As the PU scaffolds are in the 3D architecture, the cells grown into the porous scaffolds, so that it becomes hard to count the actual number in the scaffolds. However, the distribution of cells in these scaffolds is explicit. Compared with random 14%, oriented queue of cells can be clearly recognized in PU10 and PU14. Also, the number of cells in the PU14 is larger than that in the PU10.

The roles of actin in cells are driving force, skeleton, molecular motors and so on. As shown in Fig. 4C vividly, some individual L929 cells extended along the canals in the PU14. The cells in the random porous scaffolds developed lamellipodia leaning on the wall of the pore, but the ones in the aligned porous PU14 reached its lamellipodia along the canal. The formation of lamellipodia is the first step of cell migration [37]. Previous studies reported that cell motility can be altered by physical change of external environment [38, 39], and in this process lamellipodia lead cells movement [40]. It indicates the aligned 3D porous structure of PU14 can induce the oriented growth and migration of L929 cells.

To further confirm the adhesive behavior of cells, phpspho-Y397-FAK (p-FAK), an important protein in focal adhesion complex formation, was stained. Y397-FAK is adhesion-dependent and auto-phosphorylated when integrin engagement occurs [41]. FAK can collect signals from external environment and transduce them.

**Figure 5:**
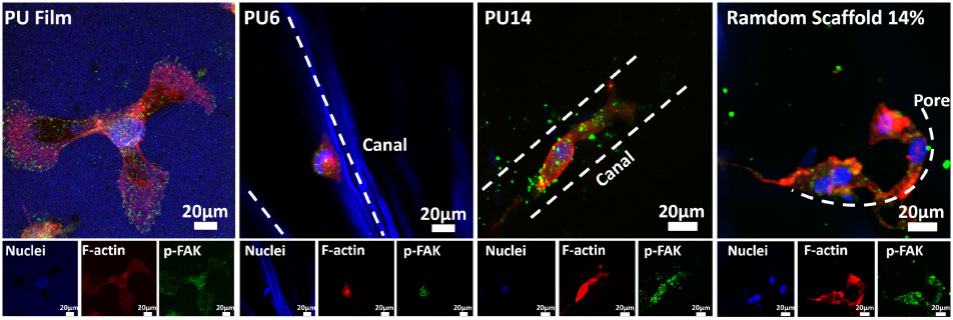
p-FAK (green) and F-actin (red) co-stained in L929 cells for PU film, aligned 3D porous PU6, aligned 3D porous PU14 and random porous scaffold of 14 wt% after culture for 8 days. Nuclei of cells were stained with dapi, blue; F-actin of cells were stained with rhodamine, red; p-FAK of cells were stained with green

Among aligned scaffolds, PU6 and PU14 were selected to perform double immunoﬂuorescent staining, because there is significant difference in the pore size and the number of cells between them. As shown in Fig. 5, the cell on the PU film can spread toward all direction. Similarly, cells on the random PU scaffold also spread well without special orientation. In contrast, the cell in the aligned porous PU14 reaches both sides of the canal to fully extend itself and grows along the canal, while cells on the aligned porous PU6 only adhere to one side of the canal.

Ray et al. hold the view that the focal adhesion of cells will be strengthened by aligned architectures [42]. At the same time, F-actin will also be stretched and become regular arrangement, because cells on aligned scaffolds crawl toward certain direction and appear to be straightened. Canals with suitable size in PU14 can constrain cells actin filament in one direction, and the tensional forces within the cytoskeleton would guide the direction of cell movement [43]. But when canals in aligned scaffolds is too wide for cells like in the PU6, cells could not reach the both side of canal which is important to obtain healthy morphology. Besides, pore wall in random porous scaffold offers a pathway for cell to claw but not to orientate. In other words, under the guide of regular canals in the PU14, cells have potentials to obtain anisotropic characters, not only on morphology but also on tendency of migration.

### Tissue response to aligned scaffolds in vivo

**Figure 6:**
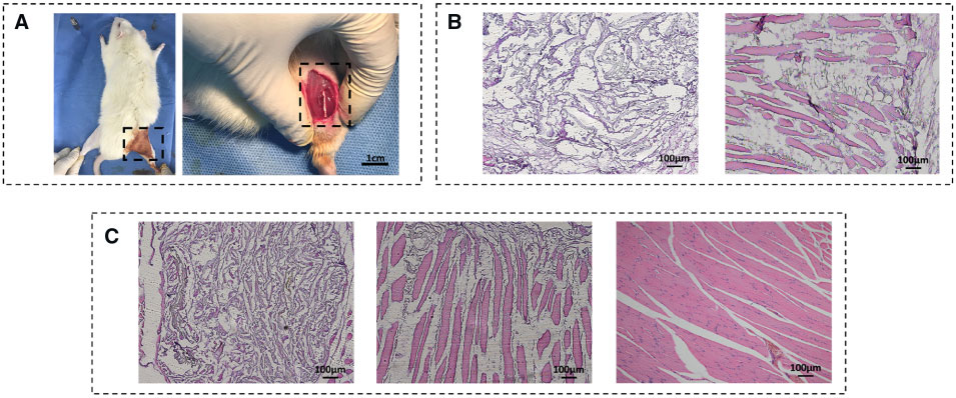
(**A**) Photograph of surgical procedure. The scaffolds were planted in the incision of 1 cm as shown in dotted box. (**B**) Picture of 1 week post-implanted regenerated muscle tissue. The scaffolds were stained into blue during H&E staining (left: random porous PU (14 wt %) scaffold, right: aligned porous PU14 scaffold). (**C**): H&E picture of 2 weeks post-implanted regenerated muscle tissue. Left: random porous PU (14 wt %) scaffold, middle: aligned porous PU14 scaffold, right: healthy muscle soft tissue

To better repair the damaged anisotropic biological tissue *in vivo*, the biological effects of the aligned scaffolds were verified in animal models. It is worth noting that the most important purpose here is to clarify the superiority of scaffolds with aligned structure in ordered tissue repair compared with random porous scaffolds, instead of exploring the best strategy of muscle repair *in vivo*. Considering the fact that the aligned porous PU14 overmatch the others for cellular response *in vitro*, the aligned porous PU14 scaffolds without any other chemical modification were selected for the implanted scaffold *in vivo* (Fig. 6). The random PU (14%) scaffolds with isotropic porous structure were used as the control because of their good ability to support cell growth *in vitro*. As shown in Fig. 6B, a mass of cells entered aligned scaffolds but few in the random one at first week. Furthermore, some regular muscle tissue could even be seen in canals of aligned scaffold. After 2 weeks post-implantation of the PU scaffolds (Fig. 6C), the scaffold nearby the natural tissue started degradation which is indicated by the fading blue-stained scaffold. Distinctively, many regular newborn tissues regenerated in the aligned PU14 scaffold while a few irregular newborn tissues appeared in random 14% scaffold. Additionally, in canal space of aligned scaffold, the isolated muscle tissue in Fig. 6B preferred connecting with each other after 2 weeks (Fig. 6C-middle) and an increase in the density of muscle tissue could be observed, so it performed more similar to the healthy leg muscle tissue as shown in Fig. 6C-right. These results indicate that the aligned 3D porous PU14 scaffold can retain its aligned feature and be able to guide the ordered tissue repair even it was partly degraded *in vivo* for 2 weeks.

**Figure 7:**
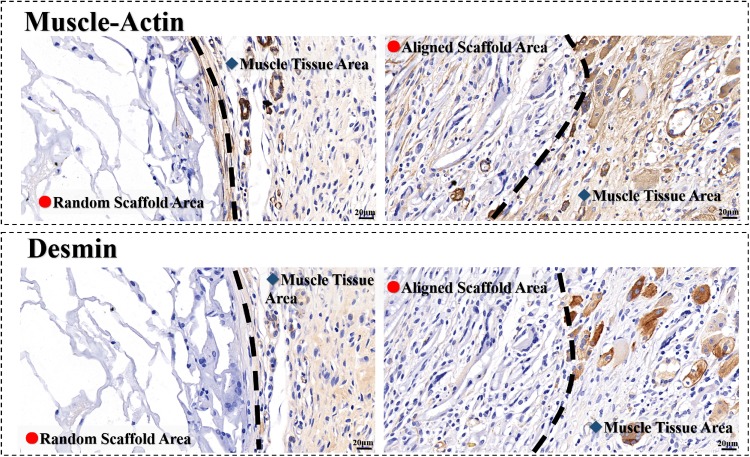
Immunohistochemical characteristics of regenerated muscle tissue in scaffolds of 2 weeks post-implanted. Left: random porous PU (14 wt%) scaffold, right: aligned porous PU14 scaffold; the color of brown at aligned scaffold areas indicate positive, indicating the newborn muscle tissues

Furtherly, muscle-actin and desmin are proteins in muscle cells. Both of them are positive in the aligned scaffold, indicating the newborn muscle tissues are presented in the scaffolds after 2 weeks post-implanted (Fig. 7). It is observed again that not only the structure but also the dense of muscle tissues in the aligned scaffold are similar to the healthy tissue around. However, in the random scaffold, few muscle tissues can be found. Most of cells in the random scaffolds distributed in the borderland. The result demonstrates that the rate of tissue regeneration in aligned scaffold is much faster than that in the random scaffold.

Both the H&E staining pictures and immunohistochemical characteristics confirm that the aligned 3D porous PU14 scaffolds can significantly accelerate the ordered tissue regeneration. In anisotropic tissue repair, random scaffold similar to porous sponge might be a complex map for cells to find entrance, but aligned scaffolds are spacious roads with a certain direction for cell to migrate in and ordered tissue regeneration.

Combining cells behaviors *in vitro* and the tissue regeneration *in vivo*, the aligned 3D porous PU14 scaffold undergoes the following procedure to favor the anisotropic tissue regeneration. Firstly, the morphology and modulus of scaffold are suitable for cells of ordered tissue to adhere and proliferate. The robust ‘fish-bone’ structure can resist the deformation *in vivo*. Also, the suitable pore size (tens of micron scale) of the scaffold permits the nutrient delivery and cells growth. Secondly, the aligned canals in 3D porous PU scaffold stimulate lamellipodia formation and tissue regeneration along the canal direction. Meanwhile, the aligned structure may induce oriented internal fluid, as descried in literature that fluid pressure could guide the cell motility [44]. Thirdly, the anisotropic scaffold can retain its aligned feature and continue to guide the ordered tissue to repair during the degradation process as presented in tissue response assessment.

## Conclusions

In this work, the aligned 3D porous PU scaffolds were successfully prepared from a green process. The aligned 3D porous scaffolds are more suitable for anisotropic tissue repair than random porous scaffolds. Even without any modification, they show availabilities of accelerating directional migration of cells and anisotropic tissue repair both *in vitro* and *in vivo*. The result of muscle repair here shows promising potentials to apply such pure material-based scaffolds with aligned structure in more other ordered tissues, such as nerve, spinal cord and so on.

## Supplementary Material

rbz031_respond_to_reviewers_Supp
