# Supplementary material for: Aligned 3D porous polyurethane scaffolds for biological anisotropic tissue regeneration
Source: Regen Biomater. 2019 Sep 30;7(1):19–27. doi: 10.1093/rb/rbz031 (PMC7233617; doi:10.1093/rb/rbz031)
Supplement: rbz031_respond_to_reviewers_Supp [file rbz031_respond_to_reviewers_supp.docx]

Dear Dr. Nicholas Peppas,

Thank you very much for your recent letter concerning our manuscript entitled “Aligned 3D porous polyurethane scaffolds for biological anisotropic tissue regeneration" (Full paper, ID RB-2019-035). According to the reviewers’ comments, this manuscript is revised carefully. The corrections are highlighted in red in the text and the response to the reviewers’ comments are presented in the answers to the reviewers.

We would like to express our sincere thanks to the reviewers’ comments and valuable suggestions. We believe the manuscript is suitable for publication in Regenerative Biomaterials.

Thank you for your consideration. We are looking forward to hearing from you.

Sincerely,

Prof. Hong Tan,

College of Polymer Science & Engineering

Sichuan University

No.24 South Section1, Yi Huan Road, Chengdu 610065, P.R. China

Phone: +86 (028)85460961

Email: [hongtan@scu.edu.cn](mailto:hongtan@scu.edu.cn)

Reviewer: 1

Comments to the Author

In this work, Tan et al. fabricated biodegradable waterborne polyurethane scaffolds porous structures through directional freeze-drying method. The study was systematically investigated. The work was wll organized. So I think it can be accepted for publication.

Comments; The porous polymer scaffold were reported by some papers, e.g. Biomacromolecules, 2014,15, 1019, Please cite them.

A: Thanks for your comments. More reports about aligned porous scaffold are added in the revised manuscript and highlighted in red.

Reviewer: 2

Comments to the Author

The authors report a green chemistry technique based on an aqueous freeze-dry process to generate aligned-pore polyurethane scaffolds for muscle tissue regeneration. Authors prepared scaffolds with varying concentrations of reagents and varying freezing media. Results were reported quantitatively for SEM alignment and tensile properties, and qualitative micrographs were provided for in vitro and in vivo studies. The in vivo image-based results are impressive but not conclusive without intermediate controls. I recommend incorporating quantitative analysis where possible in the cell studies such as quantifiable staining of actin and myosin in vitro and quantitative cell counting and reporting. Additionally, please further address clarity of language in the manuscript.

A: Thank you for your suggestions. To obtain the quantitative data of L929 cells, a great number of cells are needed. As a three-dimensional architecture of the scaffolds *in vitro*, the cells grown into the 3D porous scaffolds, so that it is hard to count the actual number in the scaffolds. Even the combined image of optically sectioned confocal microscope images of 3D scaffolds can only demonstrate the distribution of cells without details of staining colors. In fact, we try to use more confocal microscope images of each sample to count the number of cells. However, the poor identification of a single cell in the scaffold makes the statistical data unreliable. So, we qualitatively discuss the distribution of cells in micrographs instead of quantitative analysis. Some discussion on this question has added in the revised manuscript.

In order to further demonstrate the actual impact in vivo of aligned scaffolds, H&E staining and immunohistochemical staining were performed. The focus of these animal experiments is to clarify the potentials of aligned scaffolds in ordered tissue repair, instead of exploring the best strategy of muscle repair. And the result of muscle regeneration of rat showed a great improvement by using aligned scaffolds, not only in tissue arrangement but also in muscle protein.

We carefully checked the language including descriptions and grammar errors throughout the manuscript. The mistakes and inappropriate descriptions are revised and highlighted in red.

Reviewer: 3

Comments to the Author

Lin et al prepared a series of aligned three-dimensional (3D) scaffolds from biodegradable waterborne polyurethane emulsion via directional freeze-drying method. An optimized aligned PU scaffold (PU14) was selected for biological studies which facilitated the development of orientated cells and anisotropic tissue regeneration both in vitro and in vivo. The manuscript is well organized and the results are well supported by the experiments. I recommend to publish the manuscript in Regenerative Biomaterials in the current version.

A: Thanks for your comments.
